# Somatotype, Accumulated Workload, and Fitness Parameters in Elite Youth Players: Associations with Playing Position

**DOI:** 10.3390/children8050375

**Published:** 2021-05-10

**Authors:** Hadi Nobari, Rafael Oliveira, Filipe Manuel Clemente, Jorge Pérez-Gómez, Elena Pardos-Mainer, Luca Paolo Ardigò

**Affiliations:** 1 Department of Physical Education and Sports, University of Granada, 18010 Granada, Spain; 2 HEME Research Group, Faculty of Sport Sciences, University of Extremadura, 10003 Cáceres, Spain; jorgepg100@gmail.com; 3 Sports Scientist, Sepahan Football Club, Isfahan 81887-78473, Iran; 4 Department of Exercise Physiology, Faculty of Sport Sciences, University of Isfahan, Isfahan 81746-7344, Iran; 5 Sports Science School of Rio Maior—Polytechnic Institute of Santarém, Av. Dr. Mário Soares, 2040-413 Rio Maior, Portugal; rafaeloliveira@esdrm.ipsantarem.pt; 6 Research Centre in Sport Sciences, Health Sciences and Human Development, Quinta de Prados, Edifício Ciências de Desporto, 5001-801 Vila Real, Portugal; 7 Life Quality Research Centre, Av. Dr. Mário Soares, 2040-413 Rio Maior, Portugal; 8 Escola Superior Desporto e Lazer, Instituto Politécnico de Viana do Castelo, Rua Escola Industrial e Comercial de Nun’Álvares, 4900-347 Viana do Castelo, Portugal; Filipe.clemente5@gmail.com; 9 Instituto de Telecomunicações, Delegação da Covilhã, 1049-001 Lisboa, Portugal; 10 Health Sciences Faculty, Universidad San Jorge, Autov A23 km 299, 50830 Villanueva de Gállego, Zaragoza, Spain; epardos@usj.es; 11 Department of Neurosciences, Biomedicine and Movement Sciences, School of Exercise and Sport Science, University of Verona, 37131 Verona, Italy; luca.ardigo@univr.it

**Keywords:** VO_2max_, anthropometric, body composition, maturation, peak power, training load

## Abstract

The purpose of this study was three-fold: (1) to describe anthropometric, maturation, and somatotype differences of players based on playing positions; (2) to analyze variations of accumulated load training (AcL) and fitness parameters between playing positions; and finally (3) to explain the variation of maximal oxygen uptake (VO_2max_) and peak power (PP) through the AcL, body fat (BF), maturity, somatotype and fitness levels. Twenty-seven male youth soccer players under-16 were divided by the following positions participated in this study: six central midfielders, four wingers (WG), five forwards, eight defenders, and four goalkeepers (GK). They were evaluated on two occasions: pre-season and after-season. Height, sitting height, body mass, BF, girths, percentage of BF (BF%), lean body mass, maturity, somatotype, sprint test, change of direction test, Yo-Yo intermittent recovery test level 1, Wingate, PP, VO_2max_ and fatigue index were assessed. Then, AcL was monitored during training sessions. The main results revealed significant differences between player positions for maturity offset (*p* = 0.001), for BF (*p* = 0.006), BF% (*p* = 0.015), and lean body mass kg (*p* = 0.003). Also, there were significant differences for AcL and fatigue index in pre-season between player positions (*p* < 0.05). In addition, there were some significant differences in pre- and after-season for VO_2max_ and PP between player positions (*p* < 0.05). In conclusion, GK showed higher values in anthropometric, body composition variables and maturity offset compared to the other positions, while WG presented lower levels of BF. In pre-season, there were more differences by player positions for the different variables analyzed than after-season that reinforces the tactical role of the positions, and the emphasis in increased load in the beginning of the season. This study could be used by coaches, staff, and researchers as a reference for athletes of the same sex, age, and competitive level.

## 1. Introduction

Soccer has specific requirements in different competitive levels, playing positions and age categories [1]. Soccer is multifactorial and conditioned by multiple variables such as anthropometric, body composition, somatotype, physical, physiological, and soccer-specific skills [2,3]. In this sense, scientific research regarding these topics has been developed, but still provides inclusive information [4]. With special regard to young age categories, there is a need to identify the differences between young soccer players, as any category can include different chronological and biological ages [5]. Therefore, anthropometry, somatotype and some fitness parameters are necessary to know the actual state of the player.

The identification of the somatotype helps to individualize exercise training programs which can differ by positions. Furthermore, this identification facilitates an understanding of the differences in adiposity level, robustness and musculoskeletal linearity [6]. Along with somatotype, anthropometric, body composition and physiologic variables are considered main areas regarding athletes’ performance [7]. Chamari et al. [8] reinforce the finding that technical and tactical developments are influenced by morphological characteristics. In addition, the positions of youth soccer players can differ from others on body composition [9].

Soccer can be characterized by a predominant low-to-moderate intensity activity, interspaced with periods of high-intensity actions [10]. Therefore, soccer players depend on well-developed aerobic and anaerobic metabolisms to sustain the different efforts exerted in a match. Despite a predominance of aerobic activity, the most decisive skills, such as to perform a high jump, sprint or score a goal, come from the anaerobic system [11]. Therefore, soccer players need to develop different physical qualities to ensure the best performance during a match.

One of those qualities is the maximum rate of oxygen consumption (VO_2max_). This is the physiological index most widely used for measuring aerobic fitness of soccer players and can be determined in both, laboratory and field tests. It helps to clarify the level of physical fitness and if it is high, it probably prevents or reduces the risk of injury [12,13,14].

A soccer game is composed of two parts of 45 min, where the movements, when playing on the field, are very complex and varied, so players require cruising capabilities throughout the game. For this reason, to achieve excellent physical fitness, a soccer player should have a high aerobic capacity [13,15].

In addition, coaches and sport science staff usually perform internal training load quantification to avoid high levels of fatigue and to reduce high risk of illness or injury [16,17]. Also, it allows a better individual and group training periodization [18,19]. Through the rating of perceived exertion (RPE) scale, it is possible to collect considerations regarding physiological characteristics applied during training sessions [20,21].

Furthermore, the knowledge of the aforementioned variables through the season is crucial and can impact training and performance during competition. Therefore, it is important to monitor, access and compare all variables during the different phases of the season. Thus, the literature is somewhat inconclusive about establishing differences in training load, anthropometric, body composition, somatotype, physical, physiological, and soccer-specific skills for player positions in youth players. Moreover, the majority of the studies split the different variables mentioned and do not use them simultaneously.

Therefore, the aims of this study were: (i) to describe baseline anthropometric, maturation, somatotype and fitness parameters differences of players based on playing positions; (ii) to analyze variations of accumulated load training (AcL) between playing positions for periods analyzed; and finally (iii) to explain the variation of VO_2max_ and peak power (PP) through the AcL, body fat (BF), maturity, somatotype, and baseline fitness levels.

## 2. Materials and Methods

### 2.1. The Experimental Approach to the Problem

The present study consists of two parts: the first is a semi-experimental design with pre- and post-test; the second is a cohort with daily monitoring for 18 weeks in the competitive season. They practiced 5 sessions a week with one match. The team usually performed one resistance training session, one short-speed training session, agility, and small-sided games (SSG) with skill and tactical training per week. The season was divided into two equal periods of eight weeks: early-season and end-season. The first assessment was in the week before starting the league (pre-season), and the second measurement was done after the league (post-season). Players were assessed on four consecutive days. Anthropometric and body composition were assessed in one day (e.g., height, sitting height, body mass, BF, and girths) then based on this information the percentage of body fat (BF%), lean body mass (LBM), the maturity and somatotype of the players were calculated. The next day, the sprint and change of direction (COD) tests were performed. In the following day, Wingate test was performed to obtain PP and fatigue index (FI), which was considered as a criterion for assessing the anaerobic capacity of players, and on the last day, the Yo-Yo intermittent recovery test level 1 (YYIRT1) was performed to estimate the aerobic power of the players along with the calculation of the VO_2max_. All tests were performed for each participant under similar environmental conditions and in the same order. Thirty minutes after each training session, all players reported load of training, then each ‘training load’ was used with training time for calculating AcL for the two periods.

### 2.2. Participants

Twenty-seven elite soccer players, belonging to the same national under-16 team competing in the national league, were evaluated for 18 weeks during a competitive season. In total, 76 training sessions and 16 competitive matches were held. To analyze the differences between player positions, they were organized by six central midfielders (CM) with maturity offset 1.72 ± 0.18 years (yrs), four wingers (WG) with maturity offset 1.55 ± 0.17 yrs, five forwards (FW) with maturity offset 2.08 ± 0.33 yrs, eight defenders (DF) with maturity offset 1.94 ± 0.19 yrs, and four goalkeepers (GK) with maturity offset 2.38 ± 0.32 yrs. Inclusion and exclusion criteria in this study were: (i) players who participated in at least 90% of training seasons; (ii) players that did not participate in another training plan along with this study; (iii) each player who was not participating in the match during a week was practicing a separate session, without the ball or through SSG. Before starting this study, explanations about the different phases of the research were given to all participants along with their parents. Also, they were informed of the potential risks and benefits of participating in the study. The study was conducted in accordance with the Declaration of Helsinki; players and their parents given and signed their informed consent to participate in this study, which was approved by the Ethics Committee of the Sport Sciences Research Institute (IR.SSRC.REC.1399.060).

We calculated an a-posteriori estimation of sample size, accepting an alpha risk of 0.05 and a beta risk of 0.2 in a two-sided test; 4 players are necessary for each group to be recognized as statistically significant, with a minimum difference of 10.06 units between any pair of groups, assuming that 5 groups exist. The common deviation is assumed to be 3.64. A drop-out rate of 0% was anticipated.

### 2.3. Procedures

#### 2.3.1. Anthropometric and Body Composition

To measure the standing height, participants stood in the stadiometer without shoes and socks. They kept heels, hips, shoulder blades and back of the head as close as possible to the stadiometer, and then feet were placed beside each other. For sitting height, participants sat on a 50 cm bench and brought their buttocks as close as possible to the stadiometer, holding their upper body straight and placing their hands on their feet, then their heights were assessed. The distance between the highest point of the head and the bench, which was at 50 cm, was calculated as sitting height. For this measurement, portable stadiometer SECA (Model 213, Germany) was used with an accuracy of 5 mm.

For measuring maturity offset and age at peak high velocity (PHV), we used the formula: Maturity offset = −9.236 + 0.0002708 (leg length × sitting height) − 0.001663 (age × leg length) + 0.007216 (age × sitting height) + 0.02292 (Weight by Height ratio), R = 0.94, R2 = 0.891, and SEE = 0.592) and for leg length = Standing Height (cm) − Sitting height (cm) [22]. To measure weight, participants only wore one pair of sports shorts for body weight on the scale SECA (model 813, England), with an accuracy of ± 0.1 kg.

The subcutaneous fat thickness of the seven points of the body including the chest, abdomen, thigh, triceps, subscapular, suprailiac and midaxillary were calculated for body density (BD) by Jackson and Pollock method and for BD and BF% with Brozek’s formula [23]. Skin thickness was obtained by calibrating Lafayette Instrument Company (Lafayette, IN, USA) with an accuracy of 0.1 mm. All measurements were performed twice on the right side of the body, the final score recorded with the mean of two measurements. The technical standard error of subcutaneous fat measurement was performed according to previous studies [24]. Other anthropometric measurements such as girths (cm), relaxed arm, flexed arm, chest, waist, hip, upper thigh, mid-thigh, calf and abdomen were measured using the techniques provided by the International Society for the Advancement of Kinanthropometry Advance also used in previous study [25]. The technical error of measurement, inter- and intra-observer, was lower than 3% for the other variables.

#### 2.3.2. Somatotype

Body somatotype, the three-dimensional distance from a profile to the mean of all profiles (endomorph, mesomorph and ectomorph) and height to weight ratio (HWR), according to Carter and Heath [5], were calculated from anthropometric measures including height, weight, four skinfold thickness (triceps, subscapular, supraspinal, and medial calf), two epicondylar breadths (humerus and femur) and two girths (upper arm flexed and tensed, and calf). The somatotypes were plotted in agreement with previous studies [25,26] on a two-dimensional grid system somatochart using the appropriate software https://www.somatotype.org/ (accessed on 20 March 2021) (Somatotype 1.2 software). All measurements were performed by an expert with five years of background in this area. All anthropometric measurements were performed in the morning [25].

#### 2.3.3. Change of Direction Test

Players did the “modified 505 agility test” [27]. A photo-finish system recorded the time of a complete 5 m turn (2 × 5 m). All procedures were described in our previous study [28]. The best of the efforts performed was used for statistical analysis. The intra-class correlation coefficient (ICC) in this study was equal to two replicates of 0.90 in this test.

#### 2.3.4. Sprint Test

For sprint test a digital timer connected to two photocells was placed at hip height, and after 10-min specific warm-up subjects stood 70 cm before the start line. To calculate the sprint time [29], the test was performed at a distance of 30 m. The best value obtained from 3 trials was used for statistical analysis. Subjects had to rest for at least 3 min between each trial. All phases of testing were monitored by the coach. In this study, COD and sprint tests were performed with the Newtest Powertimer 300-series testing system (Tyrnävä, Finland). The ICC in this study was equal to two replicates of 0.87 in this test.

#### 2.3.5. Anaerobic Power

The Wingate test [30,31] was selected to measure anaerobic power (PP and FI). After giving a warm-up to subjects, the seat height was adjusted so that knee flexion degrees were 170–175, with leg extended fully. At first, to determine the repetition per minute (RPM), the subject began to pedal at their maximum speed for 5 s. RPM was recorded immediately from the ergometer monitor. According to the calculated value and body mass (75 g per kg of body mass), the resistance load of the test was set. The testing procedure consisted of the participants performing a 10-s countdown phase and a 30-s quick pedaling phase; all subjects were verbally encouraged to continue to pedal as fast as they could for the entire 30 s. Ultimately, the desired indicators were calculated using the Wingate power software program of the Monark model 894-E ergometer (Vansbro, Sweden). The ICC in this study was equal to two replicates of 0.94 in this test.

#### 2.3.6. Aerobic Power Test

To evaluate the aerobic power, the YYIRT1 was used and then, VO_2max_ was calculated based on the following formula: VO_2max_ (mL·kg^−1^·min^−1^) = YYIRT1 distance (m) × 0.0084 + 36.4 [32]. The ICC in this study was equal to two replicates of 0.86 in this test.

#### 2.3.7. Monitoring Accumulated Training Load

Players were monitored daily for their RPE using the CR-10 Borg’s scale, a valid and reliable scale to estimate the intensity of a session [33]. To the question “How intense was your session?” players answered in the interval of number zero for the day without training, 1 for minimum effort and 10 for maximal effort. Players provided responses 30 min after the end of the training session [12,34]. Additionally, the duration of the training sessions (in minutes) was recorded for each player. As a measure of internal load, the s-RPE was calculated by multiplying the score in the CR-10 scale by the duration of the session in minutes [35,36]. Players were previously familiarized with the scale through spending two years at the club. In this study, the AcL (for training and competition) was used for 18 weeks. These weeks of the full competitive season were divided into two periods: early-season, from week (W) 1 to W8 (includes 8 competitions and 39 practice sessions); and end-season, from W9 to W16 (includes 8 competitions and 37 practice sessions).

#### 2.3.8. Statistical Analysis

Statistical analyses were performed using SPSS (version 23.0, IBM SPSS Inc., Chicago, IL, USA) and Graph-Pad Prism 8.0.1 (GraphPad Software Inc, San Diego, California, CA, USA). The significance level was set at *p* < 0.05. Data are presented as mean and standard deviation (SD). Then, inferential tests were executed. Changes between the two in-season periods were assessed using a repeated-measures analysis of variance (ANOVA), followed by Bonferroni post hoc test for pairwise comparisons. Partial eta squared (ηp^2^) was calculated as effect size of the repeated-measures ANOVA. Besides this, a one-way ANOVA was applied to compare the different assessment variables, by playing position, in each season period. Hedge’s g effect sizes with 95% confidence interval were also calculated to determine the magnitude of pairwise comparisons for between-period comparisons. The Hopkins’ thresholds for effect size statistics were used, as follows: ≤0.2, trivial; >0.2, small; >0.6, moderate; >1.2, large; >2.0, very large; and >4.0, nearly perfect [37]. Then, multiple linear regression analysis between the percentage of change in fitness levels include VO_2max_ and PP which were calculated by this formula ([POST − PRE]/PRE TEST) × 100). The independent variables considered for multiple linear regression were AcL, BF%, maturity, somatotype, and baseline fitness levels in the soccer players. The Akaike information criterion (AIC) for each model’s regression was calculated. Multiple linear regression analysis and AIC were calculated with the R software version 4.0.2 (22 June 2020; R Foundation for Statistical Computing, Vienna, Austria). The test-retest reliability assessments, ICCs, were used. The ICC >0.7 was suitable [38]. G-Power software (University of Düsseldorf, Düsseldorf, Germany) was used for the sample size calculated with the design of the study.

## 3. Results

Table 1 shows comparisons between the different playing positions for anthropometric, maturity, body composition and somatotype variables. The most important results of one-way ANOVA showed significant differences between playing positions for maturity offset (*p* = 0.001). Hence, goalkeeper (GK) presented a significantly greater value than central midfielders (CM) (*p* = 0.007; CI95% = 0.14–1.18) and wingers (WG) (*p* = 0.002; CI95% = 0.25–1.40). Also, defender (DF) presented a significantly greater value than WG (*p* = 0.032; CI95% = 0.03–1.02). For body composition variables, it shows significant differences in (body fat) BF kg (*p* = 0.006), BF% (*p* = 0.015), and lean body mass (LBM; *p* = 0.003). Those differences were found between playing positions for BF%, where WG presented a significantly smaller BF% than CM and GK, respectively (*p* = 0.022; CI95% = −11.02–−0.59 and *p* = 0.025; CI95% = −11.98–−0.55). For LBM, GK presented a significantly greater value than CM and WG (*p* = 0.002; CI95% = 3.94–22 and *p* = 0.022; CI95% = 1.09–20.87), respectively. Further results are shown in Table 1.

**Figure 1:**
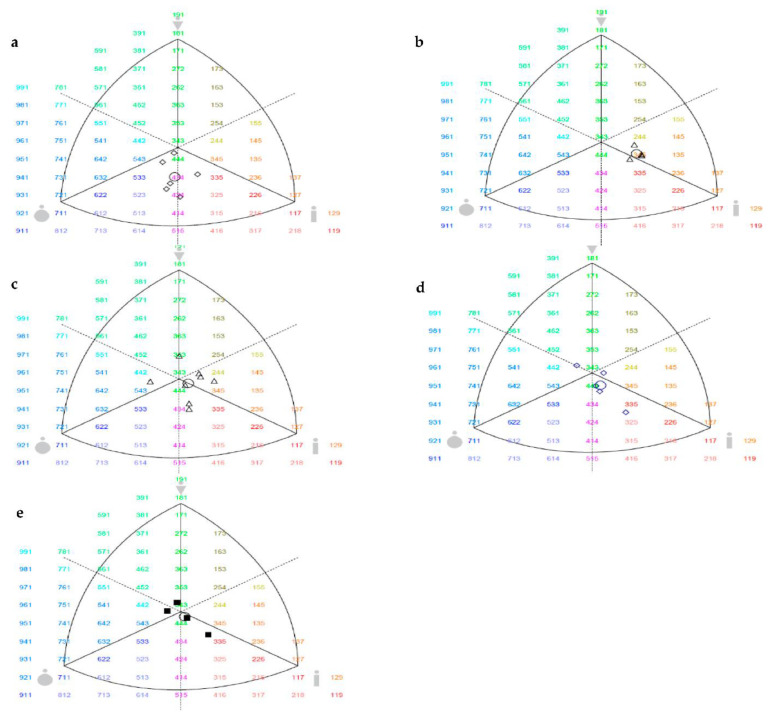
Individual somatotypes by the 2-D somatochart (**a**) Central midfielder, (**b**) Winger, (**c**) Defender, (**d**) Forward, (**e**) Goalkeeper. O = the mean somatotype.

Figure 1 shows the somatotype according to divided playing positions. The somatotype (*p* = 0.020) only showed significant differences between playing positions for endomorph where CM presented a significant greater (*p* = 0.011; CI95% = 0.29–3.15) than WG.

Significant differences between season periods in accumulated load training (AcL) demonstrated main effects of time (*F* (1, 7.73) *p* = 0.011; η_p_^2^ = 0.261) and group effect (*F* (4, 3.43) *p* = 0.025; η_p_^2^ = 0.384). Post hoc tests using the Bonferroni correction revealed a significant increase in AcL. There was an only a significant difference between early-season and end-season in forwards (FW) (*p* = 0.043; CI95% = 125.17–4942.44). Table 2 and Table 3 show comparisons between the different playing positions for fitness status in pre- and post-season, respectively. This variable was also the analysis of one-way ANOVA with a comparison between different playing position groups in each test time, and it was demonstrated that there was a difference in AcL compared to early-season (WG vs. FW: *p* < 0.021, *g* = 3.09; WG vs. GK: *p* < 0.001, *g* = 2.35; and DF vs. GK: *p* < 0.050, *g* = 1.32). However, no differences were found between playing position in end-season period for AcL.

There was a significant main effect of time for VO_2max_ (*F* (1, 10.37) *p* = 0.004; η_p_^2^ = 0.320) and group effect (*F* (4, 9.45) *p* < 0.001 η_p_^2^ = 0.632). Post hoc analysis revealed VO_2max_ was significantly greater at post-season in CM (*p* = 0.042; CI95% = 0.08–3.18) and DF (*p* = 0.048; CI95% = 0.01–1.95). Also, between player positions, this variable demonstrated that there was a significant difference within the pre-season (CM vs. GK: *p* ≤ 0.001, *g* = 4.10; WG vs. GK: *p* ≤ 0.001, *g* = 6.75; FW vs. GK: *p* < 0.002, *g* = 3.11; and DF vs. GK: *p* < 0.001, *g* = 2.57) as well as in the post-season (CM vs. GK: *p* ≤ 0.001, *g* = 3.88; WG vs. GK: *p* < 0.003, *g* = 5.66; FW vs. GK: *p* < 0.003, *g* = 2.83; and DF vs. GK: *p* < 0.001, *g* = 2.57).

Peak power (PP) levels demonstrated main effects of time (*F* (1, 22.21) *p* ≤ 0.001; η_p_^2^ = 0.502) and group effect (*F* (4, 9.12) *p* ≤ 0.001; η_p_^2^ = 0.624). Post hoc tests using the Bonferroni correction revealed a significant increase in PP between pre-season and post-season in CM (*p* = 0.024; CI95% = 19.93–183.40), DF (*p* = 0.047; CI95% = 1.11–132.39) and GK (*p* = 0.041; CI95% = 3.41–80.59). Also, between player positions, this variable demonstrated that there was a significant difference in the pre-season (WG vs. FW: *p* = 0.001, *g* = 3.93; FW vs. DF: *p* = 0.009, *g* = −1.75; and FW vs. GK: *p* = 0.001, *g* = −4.81) and ultimately, in the post-season (CM vs. FW: *p* = 0.008, *g* = 1.83; WG vs. FW: *p* = 0.002, *g* = 2.91; FW vs. DF: *p* = 0.004, *g* = −1.93; and FW vs. GK: *p* = 0.001, *g* = −3.33). Further results regarding FI are shown in Table 2 and Table 3.

**Figure 2:**
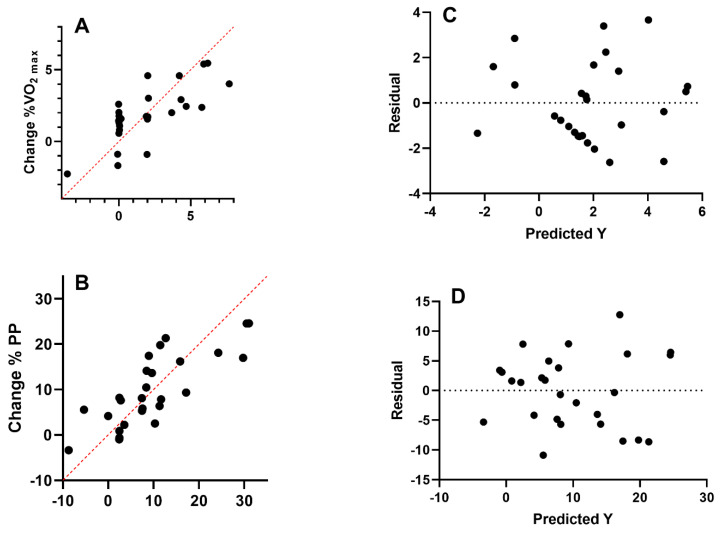
Multiple linear regression analysis was calculated to predict the percentage of change in fitness levels (**A**) VO_2max_ and (**B**) PP based on accumulated training load, body fat %, maturity, somatotype, and baseline fitness levels in the soccer players. Also, residual plot was calculated to predict the percentage of change in fitness levels (**C**) VO_2max_ and (**D**) PP; the difference between the actual value of the dependent variable and the value predicted by the residual provided. Note: VO_2max_ = maximal oxygen consumption (mL·kg^−1^·min^−1^); PP = Peak power (watts).

Multiple linear regression analysis was calculated to predict the percentage of change in fitness levels (i.e., VO_2max_ (mL·kg^−1^·min^−1^) and peak power (PP, watts)) based on AcL, BF%, maturity, somatotype, and baseline fitness levels in soccer player (Figure 2 and Table 4). The first analysis of VO_2max_ showed that there were significant (*F* (8, 18) = 2.71, *p* = 0.038), with a R^2^ of just 0.55. Participants showed good predictions for VO_2max_; (Y) is equal to Beta 0 + Beta1 (Acl) + Beta2 (BF%) + Beta3 (peak height velocity, PHV) + Beta4 (mesomorph) + Beta5 (sprint) + Beta6 (PP) + Beta7 (FI) + Beta8 (VO_2max_), where AcL was measured as A.U, PHV was measured as years, fitness status was measured as COD (change of direction, seconds), PP (watts), FI (fatigue index, %), and VO_2max_ (mL·kg^−1^·min^−1^) in order based on the equation.

There was significant statically found in PP (*F* (8, 18) = 3.80, *p* = 0.009), with an R^2^ of 0.63. Participants showed good predictions for PP; (Y) is equal to Beta 0 + Beta1 (Acl) + Beta2 (BF%) + Beta3 (maturity offset) + Beta4 (mesomorph) + Beta5 (COD) + Beta6 (PP) + Beta7 (FI) + Beta8 (VO_2max_), where AcL was measured as A.U, maturity offset was measured as years, fitness status was measured as sprint (seconds), PP (watts), FI (%), and VO_2max_ (mL·kg^−1^·min^−1^) in order based on the equation.

## 4. Discussion

The purposes of this study were: (i) to describe anthropometric, maturation, and somatotype differences of players based on playing positions; (ii) to analyze variations of accumulated load training (AcL) and fitness parameters between playing positions; and (iii) to show a multiple linear regression analysis between the percentage of change in fitness levels and variables of AcL, body fat percentage (BF%), maturity, somatotype, and baseline fitness levels. In this context, the present study contributes to the existing literature, providing information about the variables mentioned in youth athletes of a professional soccer club.

Regarding the first aim of this study, it was found that goalkeepers (GK) presented higher height, weight, maturity offset, BF and lean body mass (LBM) than other positions. Then, the wingers (WG) showed significantly less BF than GK, and the others positions as well, but the central midfielders (CM) presented lower LBM than other positions. The results are similar to those found in under-16 Spanish soccer players [39] and under-17 Brazilian soccer players [40].

The somatotype results showed some differences between player positions. For instance, the CM and GK presented high endomorph values while WG, defenders (DF) and forwards (FW) presented high ectomorph values. These results are consistent with anthropometric and body composition variables from the present study. Also, it is possible to observe some differences by player positions when compared with the study of Fidelix et al. [40], where it was found that the morphological configuration of DF, FW and GK was classified as a balanced mesomorph, while midfield players were classified as ectomorph-mesomorphs; in the present study the morphological configuration of the majority of GK and FW was classified as endomorph ectomorph, while DF and WG were considered balanced ectomorph and CM as endomorph-ectomorph. Some years ago, Rienzi et al. [41] found that GK possessed different somatotype characteristics from the other field positions. Even before, Casajús and Aragonés [42] found higher endomorph values for GK. Gil et al. [39] observed that under-16 soccer players presented higher mesomorph values (2.3-4.3-3.1), but the present study presented more balanced data. Possible explanations for the present result could be associated with food habits from Iran and some genetic influence which was not controlled in the present study.

Despite some differences between player positions, there are some findings somewhat hard to explain. From the assessment in pre-season, it was shown that WG accumulated higher training loads than other positions and GK accumulated lower training loads than other positions. In agreement with these findings, there was a lower VO_2max_ for GK, while other positions presented similar values and WG the highest VO_2max_. Also, higher fatigue index (FI) was shown for GK. However, sprint and change of direction (COD) was similar between positions, and peak power (PP) was higher for GK and WG, while FW presented the lowest value. Considering the anthropometric, somatotype, maturation and body composition assessment of the soccer players, the results are difficult to explain and it is not possible to identify a pattern for each player’s position. Previously, it was suggested that players that accumulated higher training loads required higher levels of aerobic capacity [43]. This per se, is associated with higher fat-free mass [44]. On the one hand, in the scenario of the present study, GK presented higher LBM but lower VO_2max_. On the other hand, GK presented higher BF which is in opposition to the statements of Goran et al. [44]. It is important to highlight that the results for VO_2max_ came from Yo-Yo intermittent recovery test level 1 (YYIRT1) and different results could occur with a continuous incremental and maximal test.

In the present study, the only players who showed characteristics different from other positions’ somatotype were midfield players. The distance traveled by midfield players is significantly higher than that of backs and FW [45], suggesting that this playing position requires a higher level of aerobic capacity [35], which is strongly influenced by fat-free mass [45]. Also, GK presented lower training load accumulation and higher FI, but it is suggested that higher training load accumulation should lead to higher levels of fatigue [46]. In addition, it is suggested that players with a greater intermittent aerobic capacity have reduced fatigue [47] and vice-versa, which is supported by our study. Furthermore, we also speculate that the higher values for FI could be associated with the number of impacts with soil that GK suffered.

Previously, it was reported that the magnitude of the relationships between age, maturation, body dimensions and match running performance were position dependent. Within a single age-group in the present player sample, maturation had a substantial impact on match running performance, especially in attacking players. Coaches may need to consider players’ maturity status when assessing their on-field playing performance [48]. The present study supports the mentioned findings; however, the GK presented the highest level of maturity offset and the FW the second highest level. Meanwhile, this study did not assess match running performance, but it measured AcL, which reflects internal training load perceived by the external load experienced. However, in this scenario, the GK presented the lowest values and the FW the second lowest values during pre-season, but after-season FW presented the highest values.

While Taylor [49] states that in soccer, the best players can reach VO_2max_ levels of 65–70 mL·kg^−1^·min^−1^, depending on their age, level of individual performance and position on the pitch, Slimani et al. [3] consider a wide range between 48 and 62 mL·kg^−1^·min^−1^ and specifically, by position, they reported 48.4–57.5 mL·kg^−1^·min^−1^ for GK, 53.2–62.8 mL·kg^−1^·min^−1^ for DF, 54.7–63 mL·kg^−1^·min^−1^ for CM, and 54.5–62.9 mL·kg^−1^·min^−1^ for FW. Regarding pre-season, our data seems to be in line for all FW, CM, WG and DF, which presented similar values between 48.6 and 50.1 mL·kg^−1^·min^−1^ with the exception of GK who presented the lowest value (41.5 mL·kg^−1^·min^−1^). In addition, it was shown that 55% of VO_2max_ variability and 63% of PP variability is explained by independent variables, respectively (see Table 4 and Figure 2). After-season, FW, CM, WG and DF revealed a slight increase with a range between 49.6 and 51.6 mL·kg^−1^·min^−1^, with GK presenting 41.9 mL·kg^−1^·min^−1^.

In the present study of twenty-seven under-16 elite soccer players from the Iranian League, what have been found are significant relationships between VO_2max_ and peak height velocity (PHV) and between peak power with BF and peak power with sprint (all, *p* < 0.05, Table 4);recently [28], it was suggested that the higher physical capacity allows soccer players to perform with stronger exertion, which could be expressed in the values of the chronic workload and the accumulated training monotony. The same study found a relationship between the PHV and the accumulated training monotony, and between chronic workload and physical abilities that can be expressed by VO_2max_ [28].

Due to the limited sample of our study, we suggest larger samples with the same analysis to better interpret if the other variables, such as, AcL, maturity, somatotype and baseline fitness levels can predict VO_2max_ and peak power.

Higher VO_2max_ is associated with higher performance in matches such as distance traveled, intensity, number of sprints, and the amount of player involvement with the ball [50]. Also, soccer players will have more energy to move with few limitations and will have a fast recovery without increasing fatigue substantially. The statements presented regarding VO_2max_ supported some results found in the present study during pre-season. For instance, in pre-season, the position with higher VO_2max_ was WG, which was also revealed to be the position with higher AcL and more sprints. However, they did not present higher values of PP and COD tests. Possible explanations for these differences could be associated with the use of non-specific tests to assess PP for soccer players by using a bicycle ergometer; COD testing is a skill that could be developed with special care considering a specific position. After-season, the position with higher VO_2max_ was CM, but this time it only displays higher results in COD tests while other tests analyzed present different player positions with higher values (see Table 3). Despite the possible physiological and positional adaptations that may occur during the season, a justification for the presented results could be associated with previous studies that stated that in-season training load variability is very limited, and that only minor decrements or a maintenance during the season might occur [51,52,53,54], which is in line with Malone et al. [13], who posit that it is the need to win matches that influences a possible specific peak for strength and conditioning.

In agreement with Fidelix et al. [40] study, there are some limitations regarding our participants, as they belong to the same team which per se can be associated with a specific somatotype profile of the club’s intention, its geographical location and others. Also, the small sample size and their specific team and country do not allow generalizing the present finding.

This study provides useful information regarding the AcL, anthropometric, body composition, maturity, somatotype and fitness levels such as VO_2max_, PP, anaerobic power, aerobic power, COD, sprint and YYIRT1 of a youth soccer team during different in-season periods. It provides further evidence of the value of using a combination of different monitoring and assessment measures to fully evaluate the youth soccer player across a full competitive season. Moreover, it identifies differences between player positions which allow coaches, staff, and the scientific community to analyze youth soccer player with greater knowledge. Also, for coaches, this study could provide important information to be considered when planning training sessions and/or weekly periodization. For instance, coaches can use information from RPE to produce AcL and to better understand the load perceived by young soccer players. In addition, with the information from the present study, it is suggested to include the following fitness parameters when analyzing young soccer players: PHV, body composition variables such BF, somatotype, VO_2max,_ sprint and COD tests.

In future studies, it would be interesting to replicate the present study with more teams in the same season, level of competition, or even with different age groups to better interpretation of the results. Furthermore, it would be pertinent to replicate the present study with female soccer players and different age categories in order to increase knowledge on the variables analyzed.

## 5. Conclusions

In general, GK showed higher values in anthropometric, body composition variables and maturity offset compared to the other positions. In the opposite direction, WG presented lower levels of BF. In addition, there was only one significant difference in somatotype, where DF presented a higher endomorph value than WG.

Furthermore, there were several differences in the beginning of the season and few after-season. In pre-season, AcL, VO_2max_, sprint was found to be higher for WG while COD was found to be higher for CM. PP and FI was found to be higher for GK. After-season, AcL, is similar for all positions except for the GK. VO_2max_ was found to be higher for CM. Sprint was higher for WG. COD was found to be higher for CM and GK. Still, PP and FI was found to be higher for GK. These finding reinforce the tactical role of the positions as they produce different adaptations during the season. Our multiple linear regressions support these findings because they indicated that our model explains more than 50% of all the variability of the responses.

This information is useful for coaches and professionals involved in sports, as it can be used in the process of talent selection and the development of training programs because they serve as a reference for athletes of the same sex, age and competitive level.

## Figures and Tables

**Table 1:** Absolute size characteristic, body composition, somatotype and anthropometric of soccer player by playing positions.

Characteristic		Position										*p*
		CM (*n* = 6)		WG (*n* = 4)		DF (*n* = 8)		FW (*n* = 5)		GK (*n* = 4)		
		Mean	SD	Mean	SD	Mean	SD	Mean	SD	Mean	SD	
Anthropometric	Age (yrs)	15.38	0.25	15.30	0.36	15.48	0.19	15.54	0.21	15.48	0.36	0.627
	Height (cm)	170.50 *	2.07	170.50 *	2.89	173.80 *	5.12	174.00 *	4.07	181.75	2.75	0.001 ^€^
	Weight (kg)	58.34 *	2.17	56.88 *	4.76	62.98 *	6.35	63.38 *	4.70	73.44	6.43	0.001 ^€^
	Career (yrs)	7.00	1.26	5.25	1.26	6.20	1.79	6.00	1.51	7.25	1.71	0.308
Maturations (yrs)	PHV	13.67	0.22	13.75	0.40	13.54	0.29	13.48	0.41	13.05	0.58	0.115
	Maturity offset	1.72 *	0.18	1.55 *	0.17	1.94 ^#^	0.19	2.08	0.33	2.38	0.32	0.001 ^€^
Body compositions	BF%	10.86 ^#^	2.37	5.06 *	1.00	8.73	0.63	8.71	3.56	11.32	2.95	0.015 ^€^
	BF (kg)	6.31	1.26	2.85 *	0.43	5.52	0.88	5.54	2.33	8.43	2.86	0.006 ^€^
	LBM (kg)	52.04 *	3.02	54.03 *	5.01	57.46	5.51	57.84	4.61	65.01	4.16	0.003 ^€^
Somatotype	HWR	43.97	0.28	44.46	0.43	43.74	1.17	43.83	0.46	43.36	1.03	0.306
	Endomorph	3.77 ^#^	0.52	2.05	0.49	3.06	0.36	3.03	0.96	3.33	0.83	0.020 ^€^
	Mesomorph	2.10	0.89	2.33	0.13	2.64	0.89	3.00	1.03	2.78	0.52	0.360
	Ectomorph	3.62	0.23	3.90	0.47	3.44	0.86	3.44	0.37	3.18	0.76	0.428
Girths (cm)	Relaxed arm	23.00	2.02	22.43	0.79	23.72	1.29	24.35	2.18	25.88	1.18	0.068
	Flexed arm	24.83	2.27	24.78	0.67	26.14	1.28	26.40	2.78	27.70	1.06	0.201
	Chest	78.92	4.24	77.75	3.77	81.80	2.80	77.08	20.68	87.08	3.34	0.709
	Waist	72.12	4.95	69.58	3.58	72.78	3.47	72.53	6.45	79.18	3.92	0.119
	Hip	49.77	2.76	46.53 *	0.81	50.52	2.03	51.63	4.18	54.40	4.12	0.030 ^€^
	Upper thigh	85.42	4.40	83.00 *	1.22	89.22	3.55	89.65	4.70	92.35	5.20	0.024 ^€^
	Mid-thigh	45.93	1.46	43.60	1.19	46.34	3.57	48.86	4.16	48.60	4.76	0.129
	Calf	33.17	1.37	32.28	1.23	34.64	2.18	35.43	2.13	36.75	4.19	0.057
	Abdominal	68.78	3.49	67.88	2.02	70.72	2.04	70.88	8.26	72.75	6.95	0.736

CM, central midfielders; WG, winger; FW, forward; DF, Defender; GK, goalkeeper; PHV = Peak height velocity; BF = Body fat; LBM = lean body mass; HWR = Height to weight ratio; yrs, years; SD, standard deviation. ^€^ Represents a statistically significant difference between groups to one-way ANOVA (*p* < 0.05); * Represents a statistically significant difference compared with goalkeepers (*p* < 0.05); ^#^ Represents a statistically significant difference compared with wingers (*p* < 0.05).

**Table 2:** Between-group comparisons for accumulated load and fitness parameters between playing positions in pre-season.

Variables	Groups	Mean SD		Collation	M Diff	95% CI for Diff	*p*	Hedge’s g 95% CI
AcL (A.U.)	HB	M	10,182.0	HB vs. WG	−1828.5	[−4119.6 to 462.6]	0.209	−2.39 [−4.04 to −0.8]
		SD	549.7	HB vs. FW	832.4	[−1316.8 to 2981.6]	>0.999	1.25 [−0.04 to 2.6]
	WG	M	12,010.5	HB vs. DF	−275.1	[−2192.0 to 1641.7]	>0.999	−0.23 [−1.3 to 0.8]
		SD	873.6	HB vs. GK	1897.0	[−394.1 to 4188.1]	0.170	1.49 [0.1 to 2.9]
	FW	M	9349.6	WG vs. FW	2660.9 *	[280.0 to 5041.8]	0.021 ^#^	3.09 [1.2 to 5.0]
		SD	671.3	WG vs. DF	1553.4	[−620.1 to 3726.9]	0.363	1.13 [−0.2 to 2.4]
	DF	M	10,457.1	WG vs. GK	3725.5 *	1215.8 to 6235.2]	0.001 ^#^	2.35 [0.6 to 4.2]
		SD	1405.8	FW vs. DF	−1107.5	[−3130.9 to 915.9]	>0.999	−0.86 [−2.0 to 0.3]
	GK	M	8285.0	FW vs. GK	1064.6	[−1316.3 to 3445.5]	>0.999	0.76 [−0.6 to 2.1]
		SD	1737.4	DF vs. GK	2172.1	[−1.4 to 4345.6]	0.050 ^#^	1.32 [0.01 to 2.6]
VO_2max_ (mL·kg^−1^·min^−1)^	HB	M	49.9	HB vs. WG	−0.2	[−5.1 to 4.7]	>0.999	−0.08 [−1.4 to 1.2]
		SD	2.3	HB vs. FW	1.3	[−3.3 to 5.8]	>0.999	0.46 [−0.7 to 1.7]
	WG	M	50.1	HB vs. DF	1.3	[−2.8 to 5.3]	>0.999	0.42 [−0.7 to 1.5]
		SD	1.5	HB vs. GK	8.5 *	[3.6 to 13.3]	<0.001 ^#^	4.10 [1.9 to 6.3]
	FW	M	48.7	WG vs. FW	1.4	[−3.6 to 6.5]	>0.999	0.57 [−0.8 to 1.9]
		SD	2.7	WG vs. DF	1.5	[−3.2 to 6.1]	>0.999	0.50 [−0.7 to 1.7]
	DF	M	48.6	WG vs. GK	8.6 *	[3.3 to 14.0]	<0.001 ^#^	6.75 [3.2 to 10.3]
		SD	3.1	FW vs. DF	0.0	[−4.3 to 4.3]	>0.999	0.01 [−1.1 to 1.1]
	GK	M	41.5	FW vs. GK	7.2 *	[2.1 to 12.3]	0.002 ^#^	3.11 [1.2 to 5.1]
		SD	0.5	DF vs. GK	7.2 *	[2.6 to 11.8]	0.001 ^#^	2.57 [0.9 to 4.2]
Sprint (m.s)	HB	M	3.44	HB vs. WG	−0.06	[−0.76 to 0.63]	>0.999	−0.17 [−1.4 to 1.1]
		SD	0.38	HB vs. FW	0.08	[−0.57 to 0.73]	>0.999	0.19 [−1.0 to 1.4]
	WG	M	3.51	HB vs. DF	0.03	[−0.55 to 0.61]	>0.999	0.07 [−0.9 to 1.1]
		SD	0.18	HB vs. GK	0.29	[−0.41 to 0.98]	>0.999	0.76 [−0.6 to 2.1]
	FW	M	3.36	WG vs. FW	0.14	[−0.58 to 0.86]	>0.999	0.39 [−0.9 to 1.7]
		SD	0.40	WG vs. DF	0.09	[−0.57 to 0.75]	>0.999	0.26 [−0.9 to 1.5]
	DF	M	3.42	WG vs. GK	0.35	[−0.41 to 1.11]	>0.999	1.33 [−0.2 to 2.9]
		SD	0.37	FW vs. DF	−0.05	[−0.67 to 0.56]	>0.999	−0.13 [−1.3 to 0.9]
	GK	M	3.16	FW vs. GK	0.20	[−0.52 to 0.93]	>0.999	0.52 [−0.8 to 1.9]
		SD	0.27	DF vs. GK	0.26	[−0.40 to 0.92]	>0.999	0.70 [−0.5 to 1.9]
COD (m.s)	HB	M	1.97	HB vs. WG	0.07	[−0.27 to 0.42]	>0.999	0.27 [−1.0 to 1.5]
		SD	0.22	HB vs. FW	0.13	[−0.19 to 0.46]	>0.999	0.67 [−0.6 to 1.9
	WG	M	1.90	HB vs. DF	0.06	[−0.23 to 0.35]	>0.999	0.35 [−0.7 to 1.4]
		SD	0.30	HB vs. GK	0.04	[−0.31 to 0.38]	>0.999	0.18 [−1.1 to 1.5]
	FW	M	1.83	WG vs. FW	0.06	[−0.30 to 0.42]	>0.999	0.25 [−1.1 to 1.6]
		SD	0.13	WG vs. DF	−0.01	[−0.34 to 0.31]	>0.999	−0.08 [−1.3 to 1.1]
	DF	M	1.91	WG vs. GK	−0.04	[−0.41 to 0.34]	>0.999	−0.15 [−1.5 to 1.2]
		SD	0.08	FW vs. DF	−0.08	[−0.38 to 0.23]	>0.999	−0.69 [−1.8 to 0.5]
	GK	M	1.93	FW vs. GK	−0.10	[−0.46 to 0.26]	>0.999	−0.75 [−2.1 to 0.6]
		SD	0.10	DF vs. GK	−0.02	[−0.35 to 0.30]	>0.999	−0.23 [−1.4 to 0.9]
Peak Power (w)	HB	M	699.8	HB vs. WG	−130.7	[−321.4 to 60.1]	0.440	−1.32 [−2.7 to 0.1]
		SD	104.9	HB vs. FW	168.2	[−10.7 to 347.2]	0.077	1.65 [0.3 to 3.0]
	WG	M	830.5	HB vs. DF	−38.7	[−198.2 to 120.9]	>0.999	−0.31 [−1.4 to 0.8]
		SD	53.7	HB vs. GK	−145.9	[−336.6 to 44.8]	0.261	−1.59 [−3.0 to −0.1]
	FW	M	531.6	WG vs. FW	298.9 *	[100.7 to 497.1]	0.001 ^#^	3.93 [1.7 to 6.2]
		SD	76.4	WG vs. DF	92.0	[−88.9 to 272.9]	>0.999	0.78 [−0.5 to 2.0]
	DF	M	738.5	WG vs. GK	−15.3	[−224.2 to 193.7]	>0.999	−0.34 [−1.7 to 1.1]
		SD	125.5	FW vs. DF	−206.9	[−375.3 to −38.5]	0.009 ^#^	−1.75 [−3.1 to −0.4]
	GK	M	845.8	FW vs. GK	−314.2	[−512.4 to −115.9]	0.001 ^#^	−4.81 [−7.4 to −2.2]
		SD	8.7	DF vs. GK	−107.3	[−288.2 to 73.7]	0.780	−0.94 [−2.2 to 0.3]
Fatigue index (%)	HB	M	39.4	HB vs. WG	−2.8	[−6.5 to 0.8]	0.250	−1.39 [−2.8 to 0.01]
		SD	1.7	HB vs. FW	−0.5	[−3.9 to 3.0]	>0.999	−0.23 [−1.4 to 0.9]
	WG	M	42.3	HB vs. DF	−1.9	[−5.0 to 1.1]	0.595	−1.01 [−2.1 to 0.1]
		SD	2.0	HB vs. GK	−3.8	[−7.5 to −0.2]	0.036 ^#^	−2.34 [−3.9 to −0.7]
	FW	M	39.9	WG vs. FW	2.4	[−1.5 to 6.2]	0.680	0.98 [−0.4 to 2.4]
		SD	2.2	WG vs. DF	0.9	[−2.6 to 4.4]	>0.999	0.42 [−0.8 to 1.6]
	DF	M	41.4	WG vs. GK	−1	[−5.0 to 3.0]	>0.999	−0.55 [−1.9 to 0.9]
		SD	1.9	FW vs. DF	−1.5	[−4.7 to 1.8]	>0.999	−0.69 [−1.8 to 0.5]
	GK	M	43.3	FW vs. GK	−3.4	[−7.2 to 0.5]	0.121	−1.68 [−3.2 to −0.2]
		SD	1.0	DF vs. GK	−1.9	[−5.4 to 1.6]	>0.999	−1.05 [−2.3 to 0.2]

M, Mean; diff, difference; AcL, accumulated load training; COD = change of direction; VO_2max_, maximal oxygen consumption; CM, central midfielder; WG, winger; FW, forward; DF, Defender; GK, goalkeeper; SD, standard deviation; A.U., arbitrary units; CI, confidence interval, and *p*, *p*-value at alpha level 0.05; Hedge’s g (95% CI), Hedge’s g effect size magnitude with 95% confidence interval. * The mean difference is significant at the 0.05 levels; ^#^ Indicates a significant difference between the groups with Bonferroni at the 0.05 levels.

**Table 3:** Between-group comparisons for accumulated load and fitness parameters between playing positions in after-season.

Variables	Groups	Mean SD		Collation	M Diff	95% CI for Diff	*p*	Hedge’s g 95% CI
AcL (A.U.)	HB	M	11800.2	HB vs. WG	105.9	[−3938.6 to 4150.4]	>0.999	0.05 [−1.2 to 1.3]
		SD	2087.2	HB vs. FW	−83.4	[−3877.5 to 3710.6]	>0.999	−0.04 [−1.2 to 1.2]
	WG	M	11694.3	HB vs. DF	872.3	[−2511.6 to 4256.2]	>0.999	0.45 [−0.6 to 1.5]
		SD	1307.2	HB vs. GK	3456.7	[−587.8 to 7501.2]	0.141	1.19 [−0.2 to 2.6]
	FW	M	11883.6	WG vs. FW	−189.4	[−4392.5 to 4013.8]	>0.999	−0.11 [−1.4 to 1.2]
		SD	1750.6	WG vs. DF	766.4	[−3070.6 to 4603.3]	>0.999	0.48 [−0.7 to 1.7]
	DF	M	10927.9	WG vs. GK	3350.8	[−1079.8 to 7781.3]	0.276	1.15 [−0.3 to 2.7]
		SD	1536.7	FW vs. DF	955.7	[−2616.3 to 4527.7]	>0.999	0.55 [−0.6 to 1.7]
	GK	M	8343.5	FW vs. GK	3540.1	[−663.1 to 7743.3]	0.154	1.24 [−0.2 to 2.7]
		SD	3321.4	DF vs. GK	2584.4	[−1252.6 to 6421.3]	0.474	1.07 [−0.2 to 2.3]
VO_2max_ (mL·kg^−1^·min^−1)^	HB	M	51.6	HB vs. WG	1.4	[−4.0 to 6.8]	>0.999	0.55 [−0.7 to 1.8]
		SD	2.7	HB vs. FW	1.9	[−3.1 to 7.0]	>0.999	0.61 [−0.6 to 1.8]
	WG	M	50.1	HB vs. DF	1.9	[−2.6 to 6.4]	>0.999	0.59 [−0.5 to 1.7]
		SD	1.5	HB vs. GK	9.6 *	[4.2 to 15.0]	< 0.001 ^#^	3.88 [1.8 to 5.9]
	FW	M	49.6	WG vs. FW	0.5	[−5.1 to 6.1]	>0.999	0.18 [−1.1 to 1.5]
		SD	3.1	WG vs. DF	0.5	[−4.6 to 5.6]	>0.999	0.17 [−1.0 to 1.4]
	DF	M	49.6	WG vs. GK	8.2 *	[2.3 to 14.1]	0.003 ^#^	5.66 [2.6 to 8.8]
		SD	3.2	FW vs. DF	0.0	[−4.7 to 4.8]	>0.999	0.00 [−1.1 to 1.1]
	GK	M	41.9	FW vs. GK	7.7 *	[2.1 to 13.3]	0.003 ^#^	2.83 [0.9 to 4.7]
		SD	1.0	DF vs. GK	7.7 *	[2.6 to 12.8]	0.001 ^#^	2.57 [0.9 to 4.2]
Sprint (m.s)	HB	M	3.36	HB vs. WG	−0.20	[−0.9 to 0.5]	>0.999	−0.47 [−1.8 to 0.8]
		SD	0.44	HB vs. FW	0.03	[−0.6 to 0.7]	>0.999	0.07 [−1.1 to 1.3]
	WG	M	3.55	HB vs. DF	−0.02	[−0.6 to 0.6]	>0.999	−0.05 [−1.1 to 1.0]
		SD	0.24	HB vs. GK	0.16	[−0.5 to 0.9]	>0.999	0.37 [−0.9 to 1.6]
	FW	M	3.32	WG vs. FW	0.23	[−0.5 to 1.0]	>0.999	0.62 [−0.7 to 1.9]
		SD	0.38	WG vs. DF	0.18	[−0.5 to 0.8]	>0.999	0.57 [−0.7 to 1.8]
	DF	M	3.38	WG vs. GK	0.36	[−0.4 to 1.1]	>0.999	1.19 [−0.3 to 2.7]
		SD	0.31	FW vs. DF	−0.05	[−0.7 to 0.6]	>0.999	−0.14 [−1.3 to 0.9]
	GK	M	3.20	FW vs. GK	0.13	[−0.6 to 0.9]	>0.999	0.34 [−0.9 to 1.7]
		SD	0.28	DF vs. GK	0.18	[−0.5 to 0.8]	>0.999	0.56 [−0.7 to 1.8]
COD (m.s)	HB	M	1.97	HB vs. WG	0.07	[−0.30 to 0.43]	>0.999	0.24 [−1.0 to 1.5]
		SD	0.21	HB vs. FW	0.15	[−0.19 to 0.49]	>0.999	0.75 [−0.5 to 1.9]
	WG	M	1.90	HB vs. DF	0.04	[−0.27 to 0.35]	>0.999	0.23 [−0.8 to 1.3]
		SD	0.30	HB vs. GK	0.01	[−0.36 to 0.37]	>0.999	0.04 [−1.2 to 1.3]
	FW	M	1.82	WG vs. FW	0.08	[−0.30 to 0.46]	>0.999	0.32 [−1.0 to 1.6]
		SD	0.14	WG vs. DF	−0.03	[−0.37 to 0.32]	>0.999	−0.13 [−1.3 to 1.1]
	DF	M	1.93	WG vs. GK	−0.06	[−0.46 to 0.34]	>0.999	−0.22 [−1.6 to 1.2]
		SD	0.12	FW vs. DF	−0.11	[−0.43 to 0.21]	>0.999	−0.81 [−1.9 to 0.4]
	GK	M	1.96	FW vs. GK	−0.14	[−0.52 to 0.24]	>0.999	−0.91 [−2.3 to 0.5]
		SD	0.14	DF vs. GK	−0.03	[−0.38 to 0.31]	>0.999	−0.24 [−1.5 to 0.9]
Peak Power (w)	HB	M	801.5	HB vs. WG	−55.3	[−232.0 to 121.5]	>0.999	−0.59 [−1.9 to 0.7]
		SD	103.3	HB vs. FW	206.3 *	[40.5 to 372.1]	0.008 ^#^	1.83 [0.4 to 3.3]
	WG	M	856.8	HB vs. DF	−3.8	[−151.7 to 144.2]	>0.999	−0.03 [−1.1 to 1.0]
		SD	31.2	HB vs. GK	−86.3	[−263.0 to 90.5]	>0.999	−0.94 [−2.3 to 0.4]
	FW	M	595.2	WG vs. FW	261.5 *	[77.8 to 445.3]	0.002 ^#^	2.91 [1.0 to 4.8]
		SD	102.0	WG vs. DF	51.5	[−116.2 to 219.2]	>0.999	0.55 [−0.7 to 1.8]
	DF	M	805.3	WG vs. GK	−31.0	[−224.7 to 162.7]	>0.999	−1.06 [−2.5 to 0.4]
		SD	100.6	FW vs. DF	−210.1 *	[−366.2 to −53.9]	0.004 ^#^	−1.93 [−3.3 to −0.6]
	GK	M	887.8	FW vs. GK	−292.5 *	[−476.3 to −108.8]	0.001 ^#^	−3.33 [−5.4 to −1.3]
		SD	17.7	DF vs. GK	−82.5	[−250.2 to 85.2]	>0.999	−0.90 [−2.2 to 0.6]
Fatigue index (%)	HB	M	41.3	HB vs. WG	−3.0	[−7.8 to 1.9]	0.685	−0.97 [−2.3 to 0.4]
		SD	2.3	HB vs. FW	−0.6	[−5.2 to 3.9]	>0.999	−0.24 [−1.4 to 0.9]
	WG	M	44.2	HB vs. DF	−2.1	[−6.2 to 1.9]	>0.999	−0.90 [−2.0 to 0.2]
		SD	3.5	HB vs. GK	−4.2	[−9.0 to 0.7]	0.134	−1.78 [−3.3 to −0.3]
	FW	M	41.9	WG vs. FW	2.4	[−2.7 to 7.4]	>0.999	0.73 [−0.6 to 2.1]
		SD	2.4	WG vs. DF	0.8	[−3.8 to 5.4]	>0.999	0.29 [−0.9 to 1.5]
	DF	M	43.4	WG vs. GK	−1.2	[−6.5 to 4.1]	>0.999	−0.38 [−1.8 to 1.0]
		SD	2.2	FW vs. DF	−1.5	[−5.8 to 2.8]	>0.999	−0.63 [−1.8 to 0.5]
	GK	M	45.4	FW vs. GK	−3.6	[−8.6 to 1.5]	0.379	−1.47 [−2.9 to 0.01]
		SD	1.8	DF vs. GK	−2.0	[−6.6 to 2.6]	>0.999	−0.91 [−2.2 to 0.4]

M, Mean; diff, difference; AcL, accumulated load training; COD = change of direction; VO_2max_, maximal oxygen consumption; CM, central midfielder; WG, winger; FW, forward; DF, Defender; GK, goalkeeper; SD, standard deviation; A.U., arbitrary units; CI, confidence interval, and *p*, *p*-value at alpha level 0.05; Hedge’s g (95% CI), Hedge’s g effect size magnitude with 95% confidence interval. * The mean difference is significant at the 0.05 levels; # Indicates a significant difference.

**Table 4:** Multiple linear regression analysis: percentage of change in VO_2max_ and peak power with workload, body fat, maturity, somatotype, and baseline fitness levels.

**Variables**	**Beta**	**Estimate**	**|t|**	***p* Value**	**95% CI for Estimated**	**Total Predict**
VO_2max_ (%)	β0	−42.79	2.11	0.049 *	−85.37 to −0.21	
AcL (A.U.)	β1	−0.0001	0.39	0.698	−0.0004 to 0.0003	
BF (%)	β2	0.31	1.92	0.070	−0.03 to 0.66	**R**^2^ = 0.55
PHV (years)	β3	4.41	3.41	0.003 *	1.69 to 7.12	Adjusted **R**^2^ = 0.35
Mesomorph	β4	0.30	0.49	0.628	−0.98 to 1.57	***p*** = 0.04
COD (Seconds)	β5	1.76	0.62	0.542	−4.20 to 7.72	**AIC** = 126.28
Peak power (watts)	β6	−0.002	0.46	0.649	−0.01 to 0.01	
Fatigue index (%)	β7	−0.29	0.93	0.366	−0.98 to 0.38	
VO_2max_ (mL·kg^−1^·min^−1^)	β8	−0.14	0.96	0.352	−0.45 to 0.17	
**Variables**	**Beta**	**Estimate**	**|t|**	***p* Value**	**95% CI for Estimated**	**Total Predict**
Peak Power (%)	β0	−83.58	1.11	0.279	−241.2 to 74.04	
AcL (A.U.)	β1	0.001	1.05	0.308	−0.001 to 0.002	
BF (%)	β2	1.93	2.90	0.001 *	0.53 to 3.34	**R**^2^ = 0.63
Maturity offset (yrs)	β3	6.11	0.92	0.372	−7.92 to 20.13	Adjusted **R**^2^ = 0.46
Mesomorph	β4	−3.88	1.52	0.146	−9.24 to 1.48	***p****=* 0.01
Sprint (Seconds)	β5	20.57	3.73	0.002 *	8.97 to 32.17	**AIC** = 193.12
Peak power (watts)	β6	−0.019	1.34	0.196	−0.05 to 0.011	
Fatigue index (%)	β7	−0.056	0.05	0.961	−2.42 to 2.31	
VO_2max_ (mL·kg^−1^·min^−1^)	β8	0.10	0.19	0.844	−0.97 to 1.17	

Note: β0 = Y; CI = confidence interval; AIC = Akaike information criterion; AcL = accumulated load training; BF = body fat; PHV = Peak height velocity; COD = change of direction; VO_2max_ = maximal oxygen consumption; % = The the percentage of change in between assessments from pre to post-test; A.U. = arbitrary units; yrs = years. * The significant differences at the 0.05 levels.

## Data Availability

The datasets used and/or analyzed during the current study are available from the corresponding author on reasonable request.
